# Supplementary material for: Healthcare University Courses Fail to Improve Opinions, Knowledge, and Attitudes toward Vaccines among Healthcare Students: A Southern Italy Cross-Sectional Study
Source: Int J Environ Res Public Health. 2022 Dec 28;20(1):533. doi: 10.3390/ijerph20010533 (PMC9819233; doi:10.3390/ijerph20010533)
Supplement: Supplementary file 1 [file ijerph-20-00533-s001.zip › Appendix SA.pdf]

Appendix SA

Questionnaire to future health professionals

Dear student,
the aim of this questionnaire is to collect the opinions, knowledge and attitudes towards vaccines of the healthcare students.
We ask you to take a few minutes of your time to answer the following questions.
Your answers are very important. Filling this questionnaire takes only 5 minutes.
Total confidentiality and anonymity will be maintained on the answers provided: the results will be used only for research purposes and the final report will not contain any individual data but only aggregate data.

THANKS FOR YOUR PRECIOUS COLLABORATION!

1. Give your opinion on the following statements:

	Totally disagree	Disagree	Not sure	Agree	Completely agree
I believe vaccines are important in reducing or eliminating serious diseases					
I believe vaccines are useful in certain situations, for example, in developing countries					
I don't have an opinion on this					
I believe more in natural immunity acquired through disease than in vaccines					
I don't believe in vaccinations: I think they do more harm than good					
I'm afraid of the side effects					
My religious beliefs are against vaccinations					
I don't think I'm at risk of contracting any infectious disease					
I'm afraid of getting sick after getting vaccinated					
I believe vaccines are not effective					
I am wary of the long-term health effects of vaccinations					

2. I believe health worker vaccinations are:

	Totally disagree	Disagree	Not sure	Agree	Completely agree
A prerequisite for working in the health sector					
A duty of healthcare professionals as they should be a role model for patients					

3. In your future clinical practice, would you recommend vaccinations to your patients?

☐ No ☐ Sometimes ☐ It is not within my remit ☐ Yes ☐ I don't know

3.1 If yes or sometimes, which vaccinations would you recommend most often?
………………………………………

4. Do you know which of the following vaccinations are recommended for healthcare professionals?

	Yes	No	I don't know
Seasonal flu			
Chickenpox			
MMR (measles, mumps, rubella)			
Hepatitis B			
Hepatitis A			
Tdap (tetanus, diphtheria, and pertussis for adults) or Td (tetanus and diphtheria for adults)			
Pneumococcal vaccine			
Meningococcal vaccine (tetravalent)			
BCG (anti-tuberculous)			

5. You are vaccinated for:

	No	Yes		I had the disease	I don't know
		In the last year	More than a year		
Flu					
Chickenpox					
MMR (measles, mumps, rubella)					
Hepatitis B					
Tdap (tetanus, diphtheria, and pertussis for adults) or Td (tetanus and diphtheria for adults)					

6. If you answered No to the previous question, are you planning to get vaccinated in the coming months, with:

	No	Probably	Yes	I don't know
Flu				
Chickenpox				
MMR (measles, mumps, rubella)				
Hepatitis B				
Tdap (tetanus, diphtheria and pertussis for adults) or Td (tetanus and diphtheria for adults)				

7. If you have not received any of the vaccines mentioned in question 5 please tick the possible reasons:

	Seasonal influenza vaccine	Chickenpox vaccine	MMR	Hepatitis B vaccine	Tdap or Td for adults
I did not have time					
I did not know where to go to get vaccinated					
The vaccine is not free					
The procedure is too complicated					
Other reasons					

If you answered "Other reasons", please specify them in the space below:

……

……

……

8. How do you perceive the association of specific and potential adverse effects:

	Not very probable	Medium probable	Highly probable
Flu vaccine and Guillain Barrè syndrome			
Hepatitis B vaccine and multiple sclerosis			
AS03 adjuvant of the pandemrix vaccine			

and narcolepsy			
HPV vaccine and multiple sclerosis			
Vaccine containing adjuvants and long-term complications			

9. Personal information:

Sex: ☐ Male ☐ Female Age (years): ……………………………

Degree course: ……………………………..…………………… Year of study: …………………
