# Supplementary material for: Healthcare University Courses Fail to Improve Opinions, Knowledge, and Attitudes toward Vaccines among Healthcare Students: A Southern Italy Cross-Sectional Study
Source: Int J Environ Res Public Health. 2022 Dec 28;20(1):533. doi: 10.3390/ijerph20010533 (PMC9819233; doi:10.3390/ijerph20010533)
Supplement: Supplementary file 1 [file ijerph-20-00533-s001.zip › supplementary figures.pdf]

Figures S1a -f. VH index distribution, for each degree course, by attitudes quantified using the following question "In your future clinical practice, would you recommend vaccinations to your patients?".

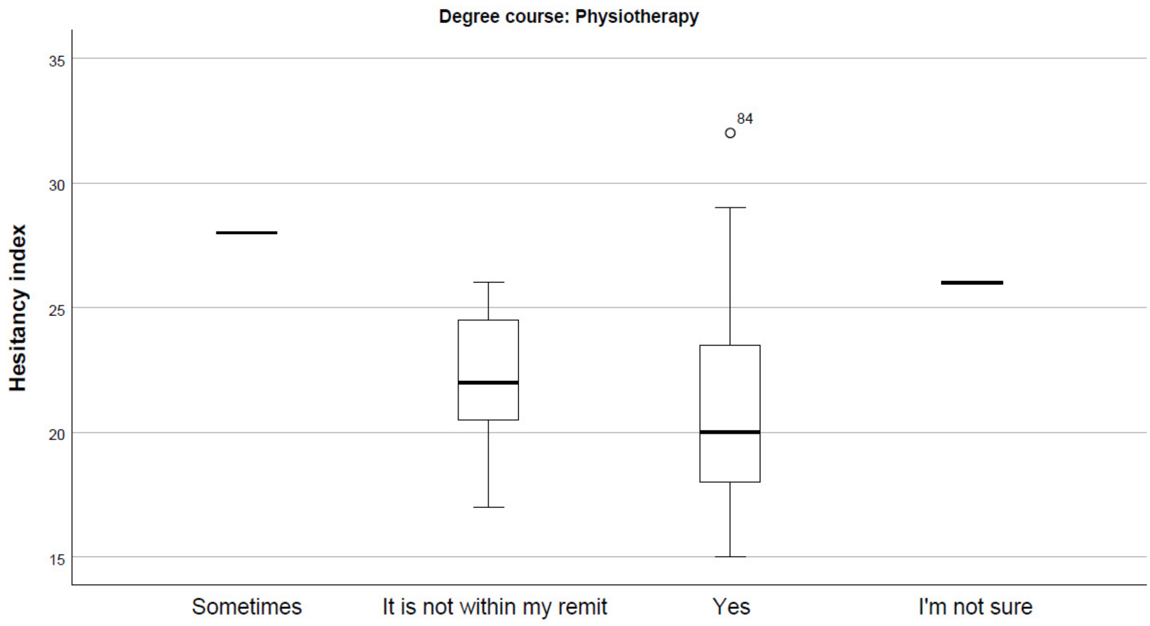

a

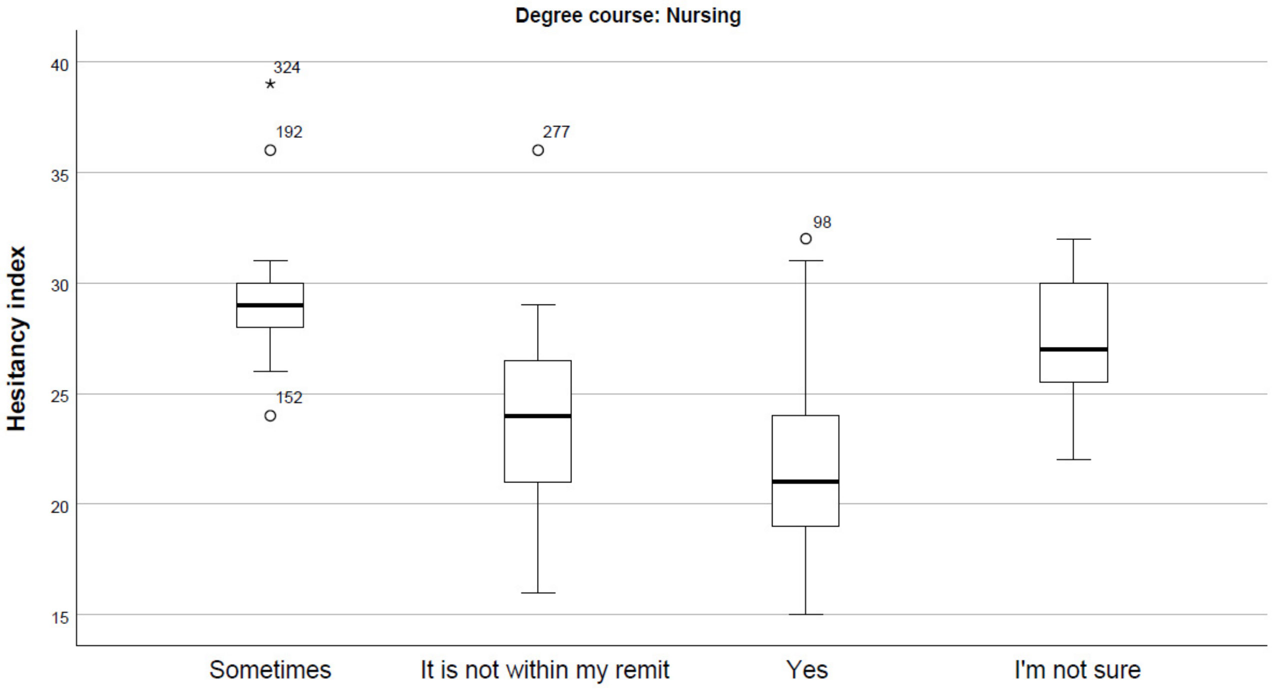

b

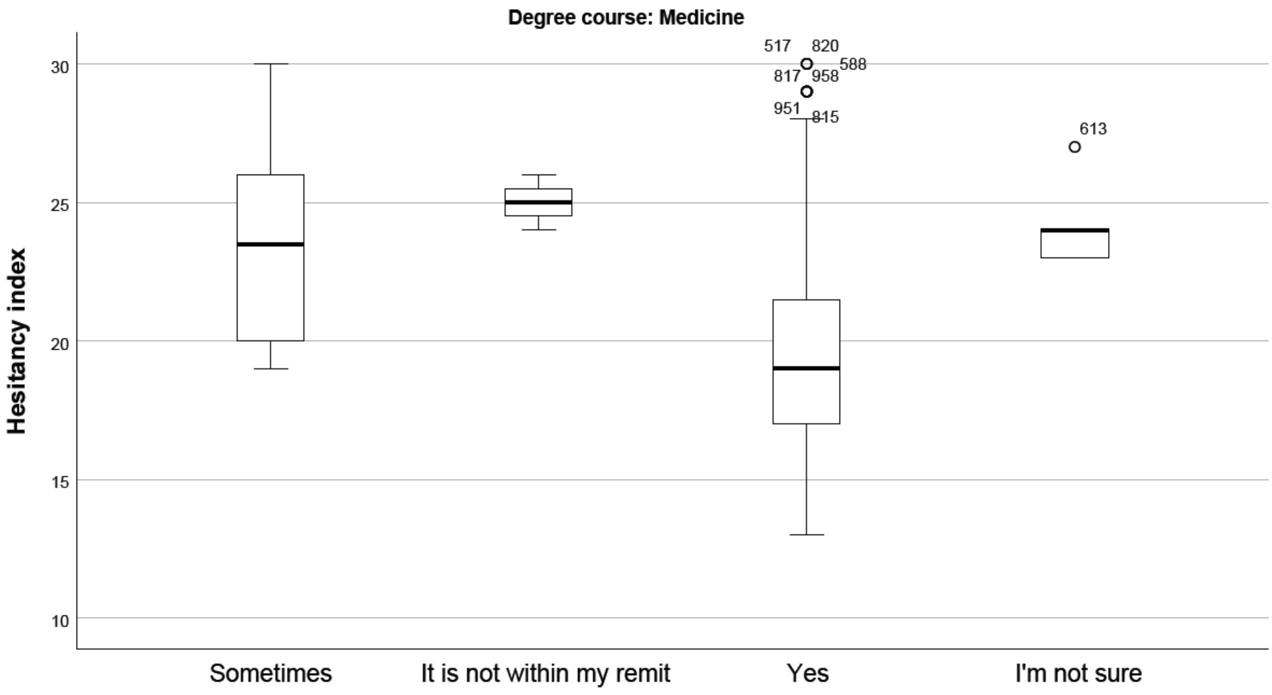
Degree course: Medicine
Hesitancy index
30
25
20
15
10
517
820
588
817
958
951
815
613
Sometimes
It is not within my remit
Yes
I'm not sure

c

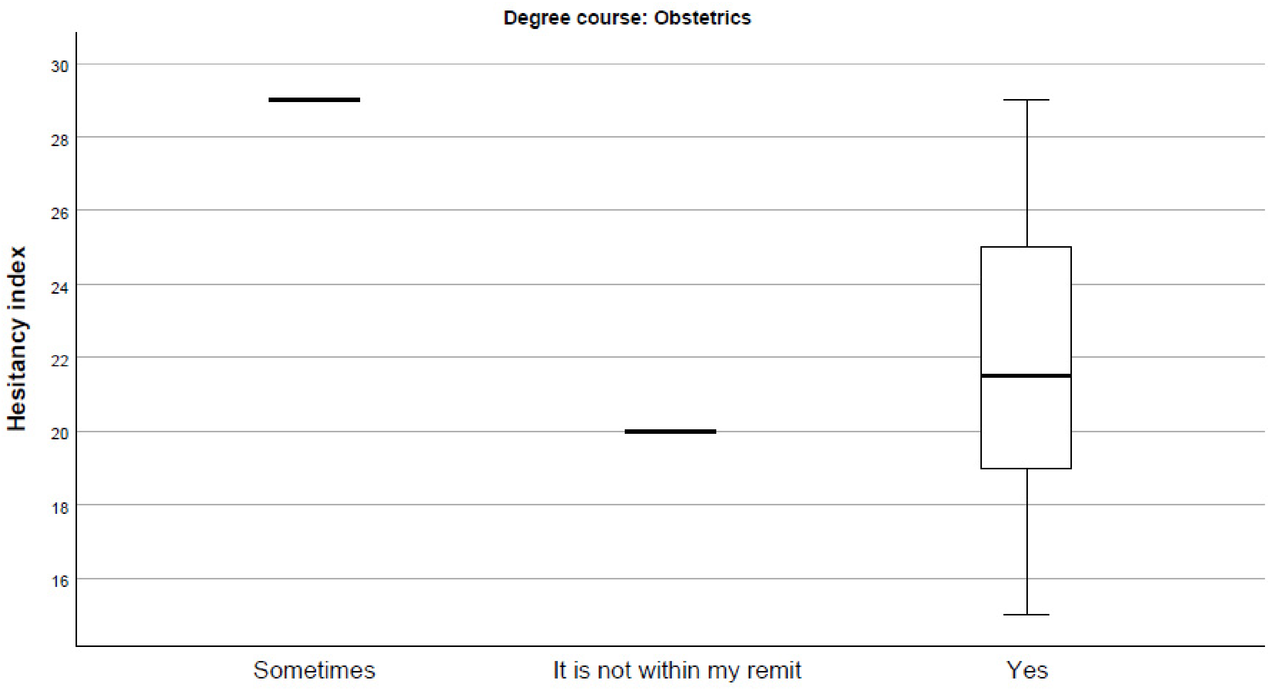
Degree course: Obstetrics
Hesitancy index
30
28
26
24
22
20
18
16
Sometimes
It is not within my remit
Yes

d

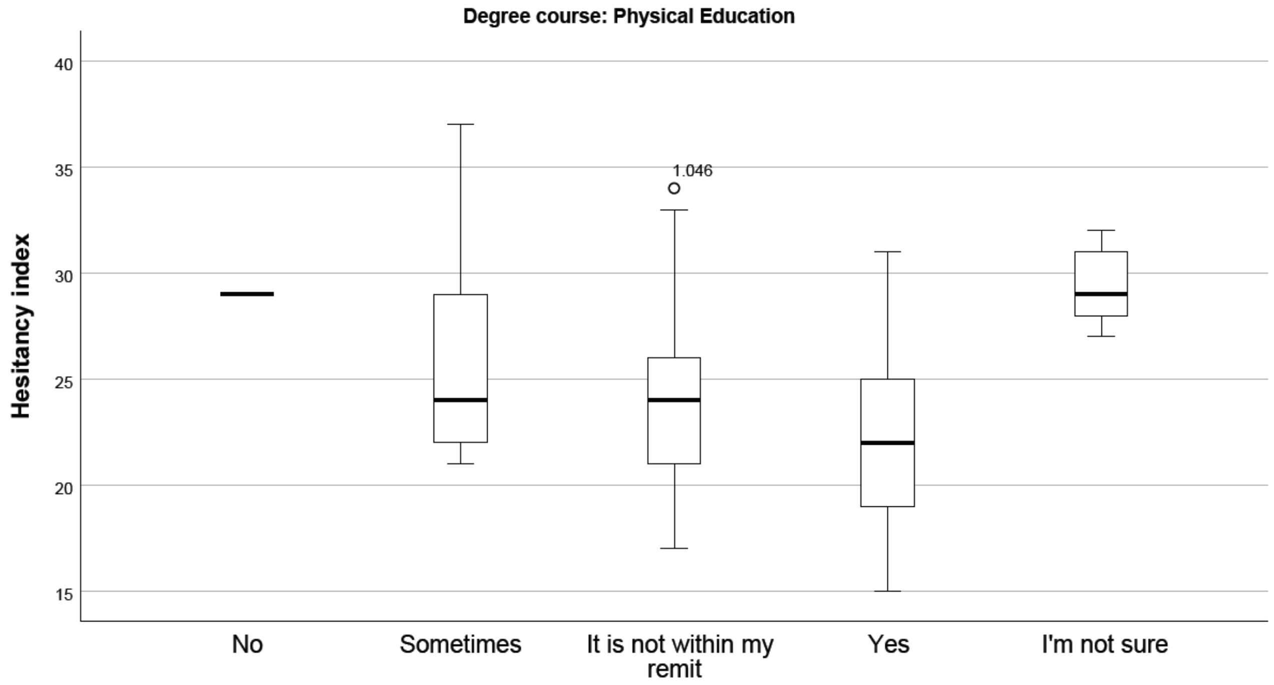
Degree course: Physical Education
Hesitancy index
40
35
30
25
20
15
1.046
No
Sometimes
It is not within my remit
Yes
I'm not sure

e

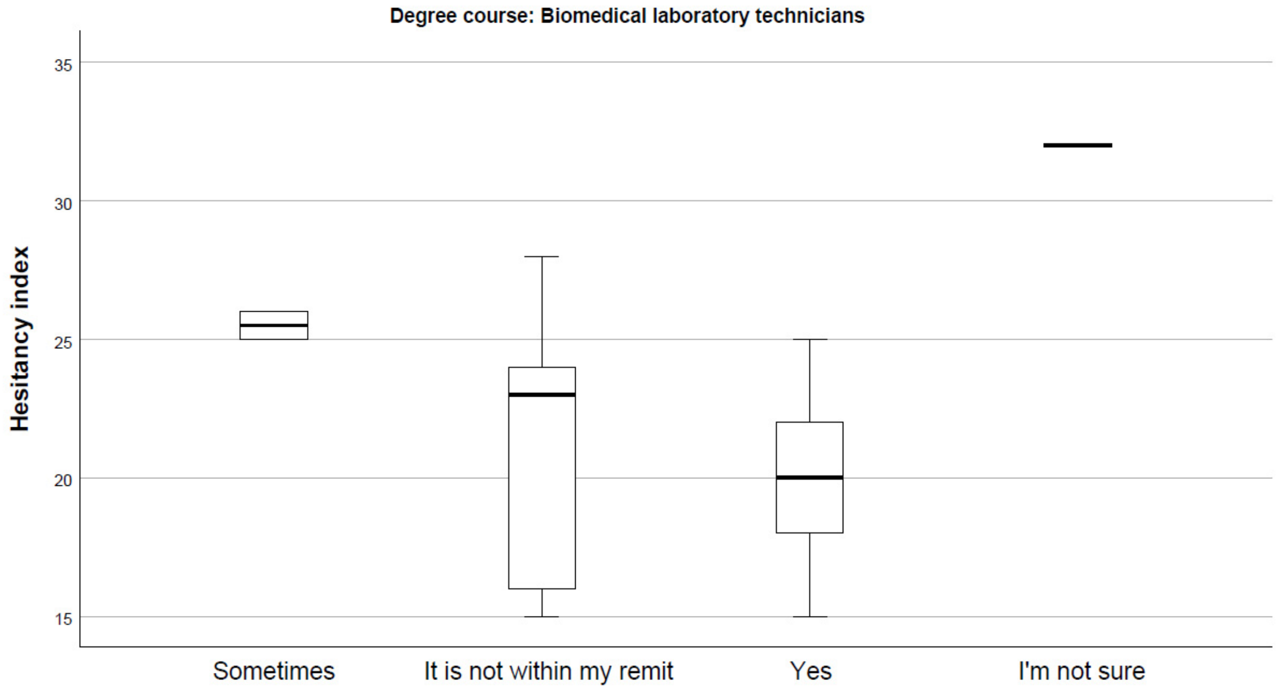
Degree course: Biomedical laboratory technicians
Hesitancy index
35
30
25
20
15
Sometimes
It is not within my remit
Yes
I'm not sure

f

Figures. S2a-c. Distribution of the vaccine hesitancy level by medical school year regarding the question "In your future clinical practice, would you recommend vaccinations to your patients?"

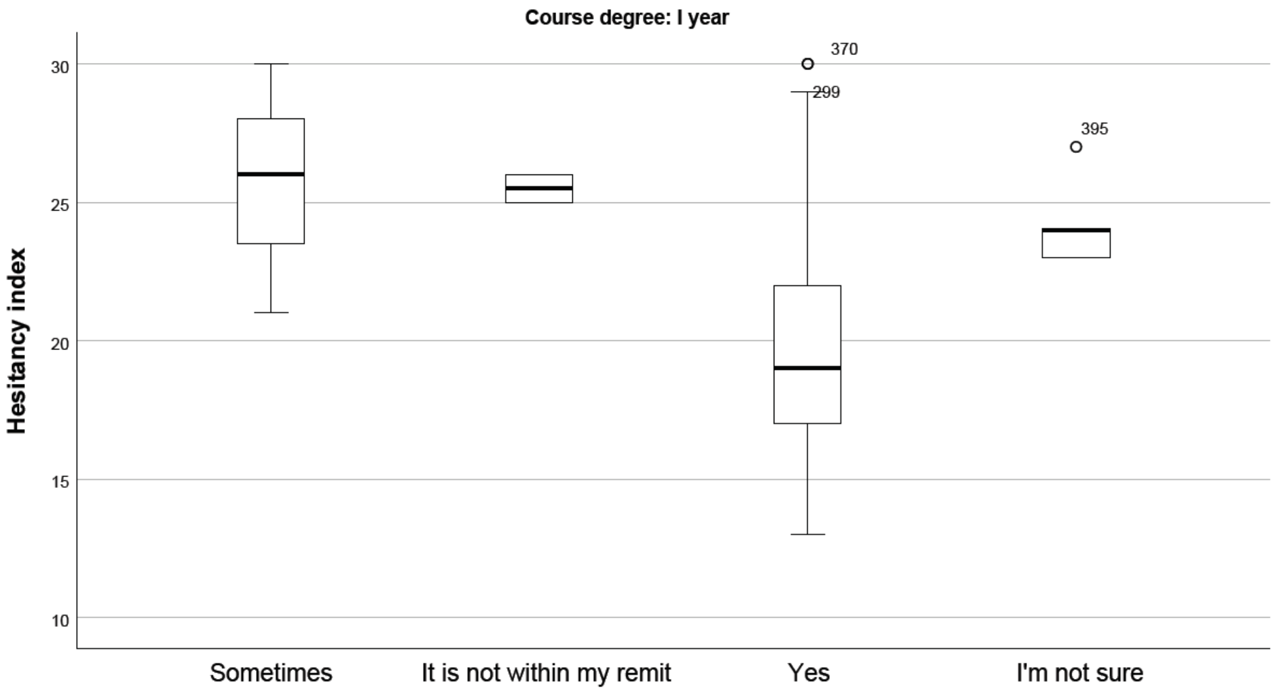

a

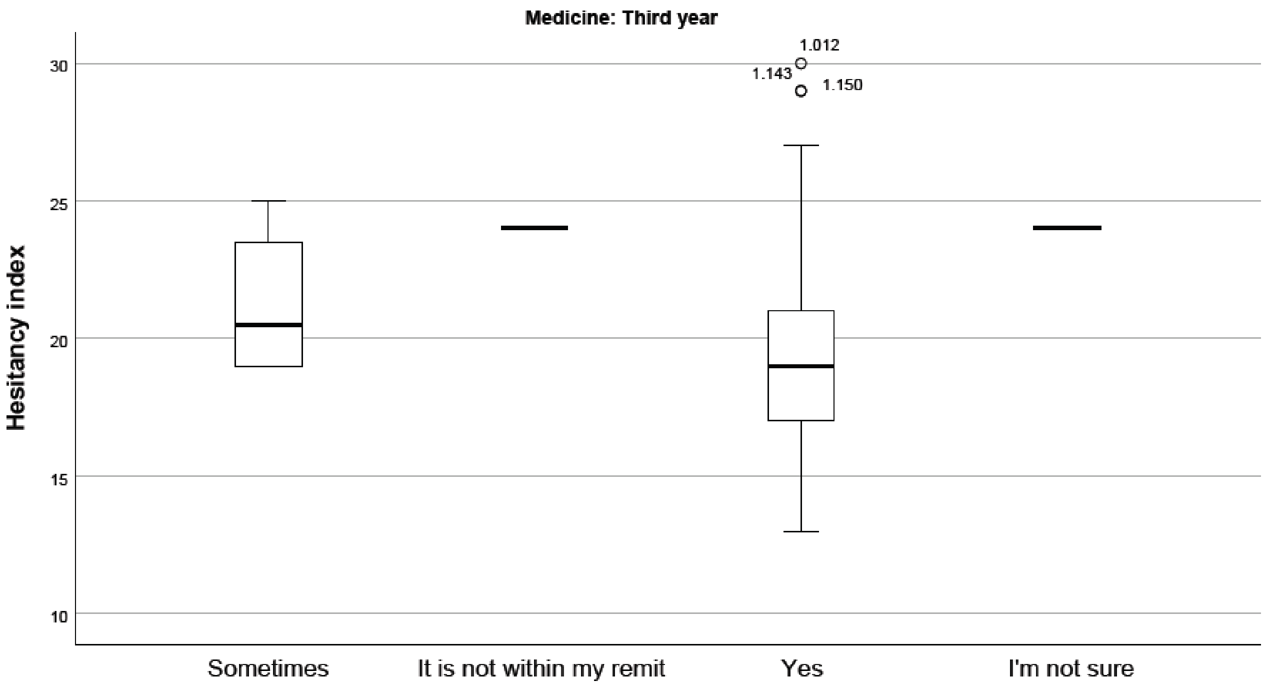

b

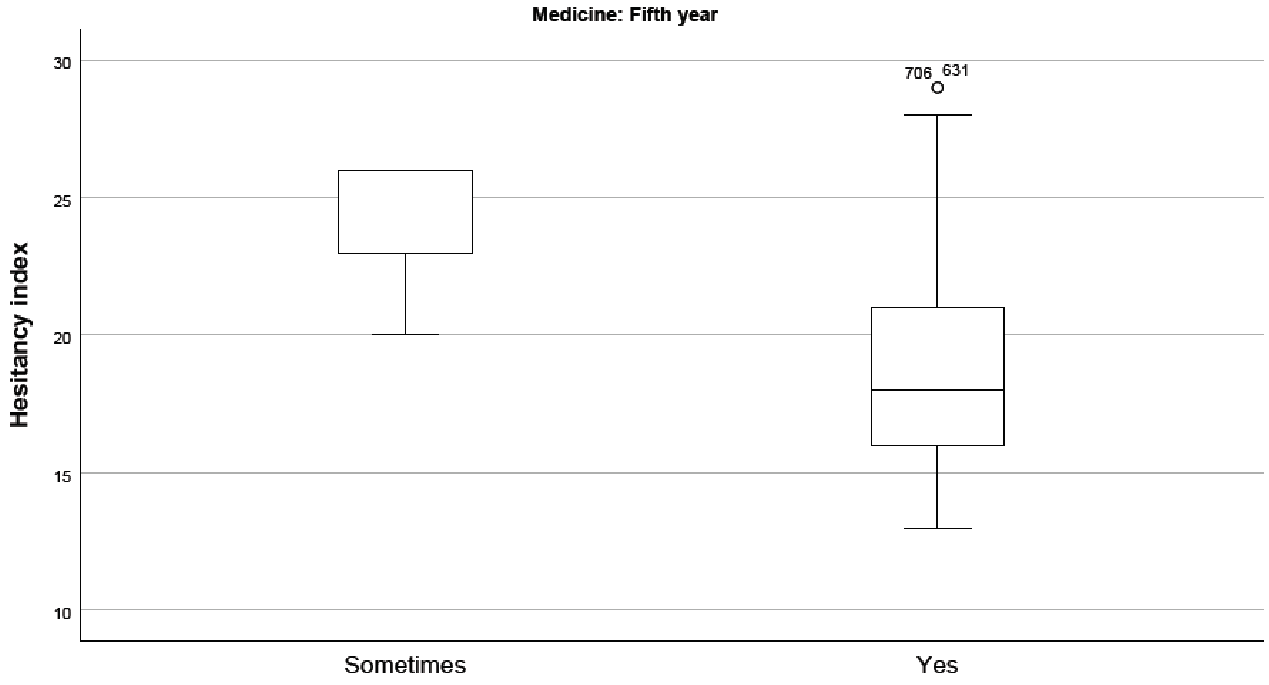
Medicine: Fifth year
Hesitancy index
30
25
20
15
10
706
631
Sometimes
Yes

C
