# Supplementary material for: Healthcare University Courses Fail to Improve Opinions, Knowledge, and Attitudes toward Vaccines among Healthcare Students: A Southern Italy Cross-Sectional Study
Source: Int J Environ Res Public Health. 2022 Dec 28;20(1):533. doi: 10.3390/ijerph20010533 (PMC9819233; doi:10.3390/ijerph20010533)
Supplement: Supplementary file 1 [file ijerph-20-00533-s001.zip › supplementary tables.pdf]

Table S1. Opinion on the following statements, by vaccine hesitancy.

Statements	Total n (%)	Vaccine hesitancy			p-value Cramers' V
		≤ 18	19 - 22	≥23	
		n (%)	n (%)	n (%)	
1. I believe vaccines are important in reducing or eliminating serious diseases	1268 (99.5)	435 (100)	437 (99.8)	396 (98.8)	0.02 (0.08)
2. I believe vaccines are useful in certain situations, for example, in developing countries	1263 (99.1)	431 (99.1)	436 (99.5)	396(98.8)	0.45
3. I don't have an opinion on this	0 (0)	0 (0)	0 (0)	0 (0)	
4. I believe more in natural immunity acquired through disease than in vaccines	55 (4.3)	0 (0)	10 (2.3)	45 (11.2)	<0.001 (0.235)
5. I don't believe in vaccinations: I think they do more harm than good	4 (0.3)	0 (0)	0 (0)	4 (1)	0.01 (0.08)
6. I'm afraid of the side effects	103 (8.1)	0(0)	18 (4.1)	85 (21.2)	<0.001 (0.33)
7. My religious beliefs are against vaccinations	0 (0)	0 (0)	0 (0)	0 (0)	
8. I don't think I'm at risk of contracting any infectious disease	76 (6.0)	2 (0.5)	28 (6.4)	46 (11.5)	<0.001 (0.19)
9. I'm afraid of getting sick after getting vaccinated	45 (11.2)	0 (0)	0 (0)	45 (11.2)	<0.001 (0.28)
10. I believe vaccines are not effective	2 (0.2)	0 (0)	0 (0)	2 (0.5)	0.1
11. I am wary of the long-term health effects of vaccinations	34 (2.7)	1(0.2)	4(0.9)	29(7.2)	<0.001 (0.19)

Notes:

We have shown in the table only the % of the column relating to the answers "agree + completely agree" therefore the % relating to the answers "Totally disagree + disagree + not sure" is equivalent to the complementary so we have not shown it in the table.

Cramer's V was entered only in case of p <0.05.

The p-value was not reported in case of cells with zero frequency.

0% means disagreeing or not sure about the statement so these are subjects who are probably not hesitant.

Table S2: Beliefs on health workers' vaccinations, by vaccine hesitancy.

Beliefs	Total n (%)	Vaccine hesitancy			p-value Cramers' V
		≤ 18 n (%)	19 - 22 n (%)	≥23 n (%)	
A prerequisite for working in the health sector	1247 (97.9)	433 (99.5)	436 (99.5)	378 (94.3)	<0.001 (0.17)
A duty of healthcare professionals as they should be a role model for patients	1189 (93.3)	424 (97.5)	419 (95.7)	346(86.3)	<0.001 (0.19)

Notes:

We have shown in the table only the % of the column relating to the answers "agree + completely agree" therefore the % relating to the answers "Totally disagree + disagree + not sure" is equivalent to the complementary so we have not shown it in the table.

Cramer's V was entered only in case of p <0.05.

The p-value was not reported in case of cells with zero frequency.

0% means disagreeing or not sure about the statement so these are subjects who are probably not hesitant.

Table S3. Knowledge distribution, by degree course.

Vaccines		**Physioterapy**			**Nursing**			**Medicine**			**Obstetrics**			**Motor science**			**Laboratorytecn.**		
		<18	19-22	>23	<18	19-22	>23	<18	19-22	>23	<18	19-22	>23	<18	19-22	>23	<18	19-22	>23
		n(%)	n(%)	n(%)	n(%)	n(%)	n(%)	n(%)	n(%)	n(%)	n(%)	n(%)	n(%)	n(%)	n(%)	n(%)	n(%)	n(%)	n(%)
Flu	Yes	8<br>36.4	9<br>25.7	7<br>25.9	33<br>58.9	65<br>61.3	48<br>43.2	192<br>62.7	116<br>54.2	73<br>57.5	1<br>16.7	9<br>52.9	4<br>22.2	17<br>50.0	35<br>59.3	48<br>45.3	6<br>54.5	4<br>57.1	4<br>33.3
	No	5<br>22.7	6<br>17.9	8<br>29.6	3<br>5.4	8<br>7.5	13<br>11.7	46<br>15.0	37<br>17.3	22<br>17.3	3<br>50.0	2<br>11.8	6<br>33.3	1<br>2.9	5<br>8.5	13<br>12.3	2<br>18.2	1<br>14.3	1<br>8.3
	I don’tknow	9<br>40.9	20<br>57.1	12<br>44.4	20<br>35.7	33<br>31.1	50<br>45.0	68<br>22.2	61<br>28.5	32<br>25.2	2<br>33.3	6<br>35.3	8<br>44.4	16<br>47.1	19<br>32.2	45<br>42.5	3<br>27.3	2<br>28.6	7<br>58.3
Chickenpox	Yes	17<br>77.3	26<br>74.3	18<br>66.7	41<br>73.2	84<br>79.2	66<br>59.5	228<br>74.5	132<br>61.7	73<br>57.5	5<br>83.3	15<br>88.2	8<br>44.4	22<br>64.7	37<br>62.2	54<br>50.9	8<br>72.7	5<br>71.4	3<br>25.0
	No	0<br>0	3<br>8.6	4<br>14.8	4<br>7.1	4<br>3.8	8<br>7.2	18<br>5.9	22<br>10.3	10<br>7.9	1<br>16.7	2<br>11.8	2<br>11.1	1<br>2.9	1<br>1.7	8<br>7.5	1<br>9.1	0<br>0.0	1<br>8.3
	I don’tknow	5<br>22.7	6<br>17.1	5<br>18.5	11<br>19.6	18<br>17.0	37<br>33.3	60<br>19.6	60<br>28.0	44<br>34.6	0<br>0	0<br>0	8<br>44.4	11<br>32.4	21<br>35.6	44<br>41.5	2<br>18.2	2<br>28.6	8<br>66.7
MMR	Yes	19<br>86.4	30<br>85.7	22<br>81.5	44<br>78.6	85<br>80.2	78<br>70.3	284<br>92.8	185<br>86.4	106<br>83.5	5<br>83.3	17<br>100.0	13<br>72.2	24<br>70.6	41<br>69.5	58<br>54.7	8<br>72.7	7<br>100.0	11<br>91.7
	No	0<br>0	0<br>0	1<br>3.7	0<br>0	4<br>3.8	3<br>2.7	1<br>0.3	3<br>1.4	3<br>2.4	1<br>16.7	0<br>0.0	1<br>5.6	0<br>0.0	1<br>1.7	4<br>3.8	0	0	0
	I don’tknow	3<br>13.6	5<br>14.3	4<br>14.8	12<br>21.4	17<br>16.0	30<br>27.0	21<br>6.9	26<br>12.1	18<br>14.2	0<br>0.0	0<br>0.0	4<br>22.2	10<br>29.4	17<br>28.8	44<br>41.5	3<br>27.3	0	1<br>8.3
Hep B	Yes	19<br>86.4	31<br>88.6	21<br>77.8	49<br>87.5	92<br>86.8	90<br>81.1	275<br>89.9	182<br>85.0	107<br>84.3	5<br>83.3	17<br>100	15<br>83.3	24<br>70.6	42<br>71.2	57<br>53.8	9<br>81.8	6<br>85.7	8<br>66.7
	No	0	0	1<br>3.7	0<br>0	0<br>0	1<br>0.9	3<br>1.0	1<br>0.5	1<br>0.8	0	0	0	1<br>2.9	0<br>0	1<br>0.9	0<br>0	0<br>0	0<br>0
	I don’tknow	3<br>13.6	4<br>11.4	5<br>18.5	7<br>12.5	14<br>13.2	20<br>18.0	28<br>9.2	31<br>14.5	19<br>15.0	1<br>16.7	0<br>0	3<br>16.7	9<br>26.5	17<br>28.8	48<br>45.3	2<br>18.2	1<br>14.3	4<br>33.3
Hep A	Yes	15<br>68.2	22<br>62.9	17<br>63.0	36<br>64.3	63<br>59.4	63<br>56.8	141<br>46.1	93<br>43.5	73<br>57.5	5<br>83.3	7<br>41.2	11<br>61.1	18<br>52.9	35<br>59.3	51<br>48.1	6<br>54.5	3<br>42.9	4<br>33.3
	No	1<br>4.5	2<br>5.7	2<br>7.4	3<br>5.4	6<br>5.7	7<br>6.3	66<br>21.6	34<br>15.9	19<br>15.0	0<br>0	1<br>5.9	2<br>11.1	1<br>2.9	0<br>0	1<br>0.9	0<br>0	0<br>0	1<br>8.3
	I don’tknow	6<br>27.3	11<br>31.4	8<br>29.6	17<br>30.4	37<br>34.9	41<br>36.9	99<br>32.4	87<br>40.7	35<br>27.6	1<br>16.7	9<br>52.9	5<br>27.8	15<br>44.1	24<br>40.7	54<br>50.9	5<br>45.5	4<br>57.1	7<br>58.3
Tdap	Yes	14<br>63.6	30<br>85.7	20<br>74.1	49<br>87.5	93<br>87.7	78<br>70.3	267<br>87.3	179<br>83.6	103<br>81.1	5<br>83.3	14<br>82.4	9<br>50.0	22<br>64.7	38<br>64.4	60<br>56.6	7<br>63.6	7<br>100.0	9<br>75.0
	No	3<br>13.6	0<br>0	0<br>0	1<br>1.8	1<br>0.9	0<br>0	3<br>1.0	4<br>1.9	5<br>3.9	0<br>0	0<br>0	0<br>0	0<br>0	1<br>1.7	0<br>0	1<br>9.1	0<br>0	1<br>8.3
	I don’tknow	5<br>22.7	5<br>14.3	7<br>25.9	6<br>10.7	12<br>11.3	33<br>29.7	36<br>11.8	31<br>14.5	19<br>15.0	1<br>16.7	3<br>17.6	9<br>50.0	12<br>35.3	20<br>33.9	46<br>43.4	3<br>27.3	0<br>0	2<br>16.7
Pneumococcal	Yes	9<br>40.9	17<br>48.6	17<br>63.0	41<br>73.2	69<br>65.1	53<br>47.7	189<br>61.8	111<br>51.9	65<br>51.2	3<br>50.0	10<br>58.8	10<br>55.6	15<br>44.1	26<br>44.1	35<br>33.0	5<br>45.5	6<br>85.7	5<br>41.7
	No	0<br>0	0<br>0	1<br>3.7	2<br>3.6	1<br>0.9	5<br>4.5	17<br>5.6	17<br>7.9	7<br>5.5	0<br>0	0<br>0	0<br>0	0<br>0	1<br>1.7	3<br>2.8	1<br>9.1	0<br>0	0<br>0
	I don’tknow	13<br>59,1	18<br>51.4	9<br>33.3	13<br>23.2	36<br>34.0	53<br>47.7	100<br>32.7	86<br>40.2	55<br>43.3	3<br>50.0	7<br>41.2	8<br>44.4	19<br>55.9	32<br>54.2	68<br>64.2	5<br>45.5	1<br>14.3	7<br>58.3
Meningococcal	Yes	11	23	16	44	78	69	244	146	86	4	14	10	18	30	36	9	6	6

		50.0	65.7	59.3	78.6	73.6	62.2	79.7	68.2	67.7	66.7	82.4	55.6	52.9	50.8	34.0	81.8	85.7	50.0
	No	0<br>0	2<br>5.7	2<br>7.4	1<br>1.8	1<br>0.9	3<br>2.7	6<br>2.0	8<br>3.7	1<br>0.8	0<br>0	0<br>0	0<br>0	1<br>2.9	0<br>0.0	2<br>1.9	0<br>0	0<br>0	1<br>8.3
	I don'tknow	11<br>50.0	10<br>28.6	9<br>33.3	11<br>19.6	27<br>25.5	39<br>35.1	56<br>18.3	60<br>28.0	40<br>31.5	2<br>33.3	3<br>17.6	8<br>44.4	15<br>44.1	29<br>49.2	68<br>64.2	2<br>18.2	1<br>14.3	5<br>41.7
BCG	Yes	12<br>54.5	19<br>54.3	11<br>40.7	44<br>78.6	72<br>67.9	63<br>56.8	201<br>65.7	134<br>62.6	81<br>63.8	3<br>50.0	13<br>76.5	10<br>55.6	20<br>58.8	28<br>47.5	37<br>34.9	6<br>54.5	5<br>71.4	6<br>50.0
	No	0<br>0	0<br>0	1<br>3.7	1<br>1.8	1<br>0.9	4<br>3.6	26<br>8.5	16<br>7.5	4<br>3.1	2<br>33.3	0<br>0	0<br>0	1<br>2.9	1<br>1.7	0<br>0	1<br>9.1	0<br>0	0<br>0
	I don'tknow	10<br>45.5	16<br>45.7	15<br>55.6	11<br>19.6	33<br>31.1	44<br>39.6	79<br>25.8	64<br>29.9	42<br>33.1	1<br>16.7	4<br>23.5	8<br>44.4	13<br>38.2	30<br>50.8	69<br>65.1	4<br>36.4	2<br>28.6	6<br>50.0

Notes: **MMR:** Measles, Mumps and Rubella. **Hep B:** Hepatitis B vaccine**. Hep A**: Hepatitis A vaccine. **Tdap:** Tetanus, Diphtheria, Pertussis, **BCG:** Bacillus Calmette-Guérin
